# Color doppler ultrasound analysis of pathological myopia induced changes in retrobulbar blood flow and its relationship with characteristic changes in myopia

**DOI:** 10.12669/pjms.39.3.7464

**Published:** 2023

**Authors:** Cui Yu, Chengcheng Xu, Zhicai Wang, Xiaohua Zhang, Xiaoming Huang

**Affiliations:** 1 Cui Yu, Department of Optometry, He Eye Specialist Hospital, Shenyang 110034, Liaoning Province, P.R. China; 2 Chengcheng Xu, School of Visual Science, He University, Shenyang 110170, Liaoning Province, P.R. China; 3 Zhicai Wang Dept. of Optometry, WenZhou Eye Valley Super Eye Hospital, Wenzhou 325000, Zhejiang Province, P.R. China; 4 Xiaohua Zhang, Department of Optometry, National Clinical Research Center for Ocular Diseases, Eye Hospital, Wenzhou Medical University, Wenzhou, 325027, China; 5 Xiaoming Huang, Department of Optometry, National Clinical Research Center for Ocular Diseases, Eye Hospital, Wenzhou Medical University, Wenzhou, 325027, China

**Keywords:** Pathological myopia, Color doppler ultrasound, Retrobulbar blood flow, Myopia

## Abstract

**Objective:**

To analyze changes in retrobulbar blood flow in patients with pathological myopia using color doppler ultrasound (CDU), and to explore the relationship of these changes with the characteristic changes resulting from myopia.

**Methods:**

One hundred and twenty patients who met the selection criteria in the ophthalmology department of He Eye Specialist Hospital from May 2020 to May 2022 were included in this study. Patients with normal vision (n=40) were considered Group-A, patients with low and moderate myopia (n=40) were considered Group-B, and patients with pathological myopia (n=40) were considered Group-C. All three groups underwent ultrasonography. The peak systolic blood flow velocity (PSV), end-diastolic blood flow velocity (EDV), and resistance index (RI) of the ophthalmic artery, central retinal artery, and posterior ciliary artery were recorded and compared, and the characteristics of these parameters and myopia severity were analyzed.

**Results:**

Pathological myopia resulted in significantly lower PSV and EDV of the ophthalmic artery, central retinal artery and posterior ciliary artery and higher RI values than patients with normal vision and low/moderate myopia (P<0.05). Pearson correlation analysis showed that retrobulbar blood flow changes were significantly correlated with age, eye axis, best corrected visual acuity, and retinal choroidal atrophy.

**Conclusion:**

CDU can objectively evaluate the retrobulbar blood flow changes in pathological myopia, and such blood flow changes are significantly correlated with the characteristic changes of myopia.

## INTRODUCTION

Myopia is one of the most common ophthalmic diseases. Further development of this disease can cause the gradual growth of the eye axis, and mechanical expansion of the eyeball wall, leading to pathological myopia. This can result in posterior scleral staphyloma, retinal choroid atrophy, a severe reduction of the patient’s vision, or even the complete loss of vision.^1^,^2^ As for the occurrence and development of pathological myopia, the current mechanism is not completely clear, but some studies have shown that it is closely related to insufficient blood supply to the eyeball, and ocular hemodynamic changes can play an important role in pathological myopia.^3^ Patients with pathological myopia have excessive axial extension and bulbar wall expansion, and there may be degenerative changes in the fundus, which may be manifested as hardening of the choroidal vessels, microthrombosis, or choroidal atrophy. As such, it is very important to understand the changes of blood flow behind the eyeball in pathological myopia.^4^

CDU is a commonly used method to examine the blood flow status of blood vessels in the clinic. This method measures the blood flow supply intensity of blood vessels as well as tissue blood flow perfusion which can be used to analyze blood flow changes.^5^,^6^ In this study, CDU was used to examine blood flow changes within the eyeball of patients with pathological myopia, in comparison to healthy patients and those with low to moderate myopia.

## METHODS

**Fig.1:**
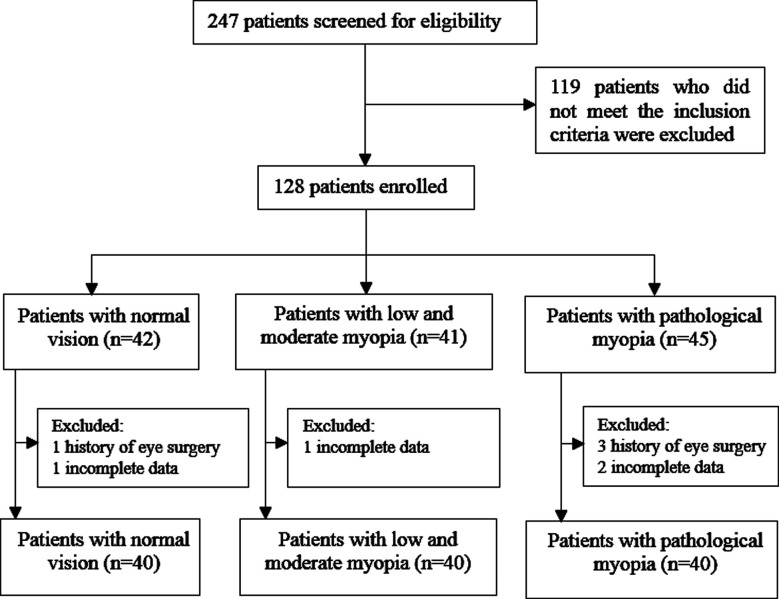
Flowchart of patient enrollment.

One hundred and twenty patients met the selection criteria in the Eye Specialist Hospital from May 2020 to May 2022 were included in this study. Patients with normal vision (n=40) were considered Group-A, patients with moderate and low myopia (n=40) were considered Group-B, and patients with pathological myopia (n-=40) were considered Group-C (Fig.1).

### Inclusion criteria:

- Patients were allocated to their respective groups in accordance with the standards proposed by the International Institute of myopia.
- Group-A excluded myopia, Group-B was diagnosed as having moderate and low myopia, and Group-C was diagnosed as having pathological myopia.
- All patients must be 18 years or older.

### Exclusion criteria:

Patients were excluded if they:

- Were diagnosed with congenital eye disease or other eye diseases
- Had a history of eye surgery
- Patients with severe cardiac, hepatic and pulmonary insufficiency
- Were diagnosed with mental health issues
- Were diagnosed with a malignant tumor

### Ethical Approval:

The medical ethics committee of our hospital approved the study (No. 2022-002-K-1-01, Date: 2022-10-02).

### Specialist examination:

The Diopter inspection was conducted with the full-automatic optometer (Nidek, Japan) to determine the nature and degree of refraction. Subjective optometry was then completed with the comprehensive optometer (Nidek RT3100) to convert the power of the isospherical mirror using this equation: the reading of the isospherical mirror = the subjective optometric spherical mirror + astigmatism / 2.^7^ Intraocular pressure was measured using a non-contact tonometer (TOPCON, CT-80a, Japan), and each eye was measured three times with the average value taken. Based on the optometry results the correct way to wear spherical and column lens was determined and measured through the international standard logarithmic vision chart. After mydriasis treatment with medori eye drops (Shentian pharmaceutical, J20180051), the fundus examination was performed through a 90D front mirror, and fundus photography was completed in high myopia mode using a fundus color camera (Chongqing Kanghua Ruiming, AOS-AER). The macular optical coherence tomography was performed using frequency domain coherence tomography technology (Spectralis, Heidelberg, Germany) to quickly perform macular scanning. The scanning line length was 6mm and the included angle was 30°.

### Ultrasonic examination:

The patient was instructed to lie on their back and to gently close their eyelids. The coupling agent was applied to the ultrasound probe, and the eyeball was scanned both horizontally and vertically. Then lens was located in the middle line of the image, with the orbital apex behind the eyeball. The orbital bone was echoed on both sides and the cross-sectional view of the optic nerve was clearly displayed. The image was frozen to allow for the observation and measurement the blood flow behind the eyeball, using CDU (Yum! MyLab 90) with a probe frequency of 7.4MHz. The central retinal artery and central vein were observed within the “V” shaped dark area of the optic nerve and 0.5cm of the posterior bulbar wall. The posterior ciliary artery was visualized on both sides of the 3-5cm posterior optic nerve dark area. The main ophthalmic artery was observed at the 10-15cm posterior orbital location and vessels above were sampled with a sampling volume of 2mm. The included angle between the blood flow and the sampling line was controlled below 20°. The best blood flow spectrum of 4-6 beat cycles was captured. The peak systolic blood flow velocity (PSV), end diastolic blood flow velocity (EDV) and resistance index (RI) of each artery was measured, where RI = psv-edv / PSV.^8^

### Observation index:

PSV, EDV and RI of the ophthalmic artery, central retinal artery and posterior ciliary artery was measured in all three groups. The correlation between the changes in blood flow behind the eyeball and the characteristic changes observed in myopia were compared.

### Statistical analysis:

It was estimated that at least 37 patients per group would be needed to provide 80% power with an effect size of 0.30, a two-sided significance level of 0.05 as measured by the R package ’pwr’ (Version 1.0.143, RStudio Inc., Boston, MA, USA). SPSS v16.0 (SPSS Inc., Chicago, IL, USA) was used for all statistical testing. Categorical variables were compared via Fisher’s exact test or Chi-squared tests. Continuous variables are means±SD (standard deviation) and were compared via Student’s t-tests or Mann-Whitney’s U tests. Correlation was analyzed by Pearson correlation. Significance was determined by *p* < 0.05.

## RESULTS

There was no statistical difference (P > 0.05; Table-I) between the three groups in terms of gender, age and other baseline characteristics. The PSV and EDV of the ophthalmic, central retinal and posterior ciliary arteries in Group-C were significantly lower than those in Group-B and A, and the RI value was higher than those in Group-B and A (P < 0.05; Table-II). Pearson correlation analysis showed that the changes of blood flow behind the eyeball were significantly correlated with age, ocular axis, best corrected visual acuity and retinal and choroidal atrophy, as shown in Table-III.

**Table-I:** Baseline characteristics of the three groups.

Group	n	Gender (Male/Female)	Age (years)	Intraocular pressure (mmHg)	BMI (kg/m^2^)	Mean arterial pressure (mmHg)
Group-A	40	21/19	32.45±5.34	13.82±2.33	23.45±1.35	85.69±4.32
Group-B	40	19/21	32.48±5.32	13.90±2.35	23.42±1.37	85.70±4.34
Group-C	40	11/18	32.42±5.35	13.92±2.32	23.46±1.33	85.67±4.36
*F*	-	0.200	0.025	0.192	0.033	0.021
*p*	-	0.645	0.980	0.847	0.973	0.984

**Table-II:** Comparison of PSV, EDV and RI in the ophthalmic artery, central retinal artery and posterior ciliary artery between the three groups (*χ̅*±*S*).

Group (n)	Ophthalmic artery			Central retinal artery			Posterior ciliary artery		

	PSV (cm/s)	EDV (cm/s)	RI	PSV (cm/s)	EDV (cm/s)	RI	PSV (cm/s)	EDV (cm/s)	RI
Group-A (n=40)	32.56±4.35	8.54±1.23	0.70±0.23	12.87±2.33	3.75±0.65	0.70±0.15	15.05±2.31	4.33±0.76	0.69±0.11
Group-B (n=40)	32.18±4.29	8.50±1.25	0.71±0.21	12.85±2.35	3.71±0.64	0.71±0.16	14.56±2.28	4.28±0.74	0.70±0.12
Group-C (n=40)	29.19±3.98^ab^	7.63±1.18^ab^	0.75±0.13^ab^	7.36±1.03^ab^	1.96±0.45^ab^	0.74±0.12^ab^	8.25±1.11^ab^	2.11±0.42^ab^	0.74±0.10^ab^
F	5.632	7.532	4.987	6.821	5.972	5.021	5.483	6.988	4.782
p	0.000	0.000	0.000	0.000	0.000	0.000	0.000	0.000	0.000

***Note:*** ^a^ represents the comparison with Group-A, P<0.05; ^b^ represents the comparison with Group-B, P < 0.05.

**Table-III:** Correlation between changes in blood flow behind the eyeball and the characteristic changes induced by myopia.

Parameter	Age		Ocular axis		Equivalent spherical mirror		Best corrected vision		Retinal choroidal atrophy	

	r	p	r	p	r	p	r	p	r	p
***Ophthalmic artery***										
PSV	-0.041	0.045	-0.012	0.037	-0.341	0.008	-0.532	0.000	-0.532	0.000
EDV	-0.712	0.000	-0.521	0.00	-0.398	0.001	-0.652	0.000	-0.762	0.000
RI	-0.881	0.000	-0.121	0.021	-0.423	0.000	-0.462	0.000	-0.678	0.000
***Central retinal artery***										
PSV	-0.063	0.042	-0.021	0.025	-0.032	0.006	-0.762	0.000	-0.521	0.000
EDV	-0.689	0.000	-0.482		-0.462	0.000	-0.659	0.000	-0.723	0.000
RI	-0.876	0.000	-0.021	0.018	-0.582	0.000	-0.702	0.000	-0.668	0.000
***Posterior ciliary artery***										
PSV	-0.121	0.040	0.020	0.026	0.030	0.004	0.756	0.000	-0.533	0.000
EDV	-0.652	0.000	-0.047	0.024	-0.498	0.000	-0.687	0.000	-0.713	0.000
RI	-0.872	0.000	-0.023	0.016	-0.567	0.000	-0.712	0.000	-0.675	0.000

## DISCUSSION

In this study, CDU was used to detect the changes in posterior ocular blood flow in patients with pathological myopia. We found that the PSV and EDV in the ophthalmic, central retinal and posterior ciliary arteries in patients with pathological myopia were significantly lower than those with moderate to low myopia or healthy vision. The RI value was significantly higher in patients with pathological myopia than those with moderate to low myopia or healthy vision (P<0.05). Previous work by Mrugacz M et al.^9^ also showed that the deterioration of blood flow parameters within the central retinal artery and short posterior ciliary artery in myopic patients led to increased severity of retinal degeneration. It is suggested that the blood supply from the ophthalmic artery, central artery and ciliary artery in patients with pathological myopia is reduced, causing insufficient blood perfusion distally, which may lead to increased atrophy of the retinal choroid and further damage to vision.^10^ Pathological myopia causes the ocular axis to be lengthened, a narrowing of the lumen of both the retinal artery and retinal vein, and an increase in the angle of the branch vessels. Therefore, PSV and EDV are decreased, while RI is increased.^11^ However, Bryl A et al 12 found that the severity of retinal degeneration in different stages of myopia is related to ocular artery blood flow, but it was not statistically significant, which may be related to race or region. It may be that a thorough understanding of the blood supply of a patient’s eyes could improve pathological myopia and degeneration by delaying the process of ocular axis extension, so that further damage to a patients’ vision can be alleviated.

A large number of clinical studies have shown that the occurrence of pathological myopia is closely related to factors such as age, ocular axis and best corrected vision, with retinal choroidal atrophy being the primary influencing factor for the further development of the disease.^13^,^14^ This study analyzed the relationship between the changes in posterior ocular blood flow and the characteristic changes of myopia. The results showed that the changes in posterior ocular blood flow were significantly correlated with age, ocular axis, best corrected visual acuity and retinal choroidal atrophy. It can be inferred that under the influence of congenital and acquired factors, the eyeball wall of patients with pathological myopia may be altered, resulting in further expansion of the eyeball volume, the extension of the ocular axis, the increase of the diopter, and the change retinal and choroid structure. All of these changes can result in the thinning of the blood vessel of the choroid and decreased function of the choroidal artery. Such changes can lead to abnormal blood circulation and bleeding of the choroid ultimately reducing total perfusion.^15^,^16^ Additionally, the microcirculation of the retina and choroid may be affected after expansion of the bulbar wall in patients with pathological myopia, which may lead to choroid atrophy. Therefore, improvements in blood flow behind the eyeball in patients with pathological myopia may provide a protective effect on their eyesight.

### Limitations:

It includes sample size was small resulting in the lack of large-scale multivariate linear analysis. As such, the results presented here may not represent all patients with pathological myopia. The conclusion may be considered subjective, and a larger-scale study should be completed to verify the relationship between changes in blood flow behind the eyeball of patients with pathological myopia and the characteristic changes associated with myopia.

## CONCLUSION

CDU can objectively evaluate the changes in blood flow behind the eyeball in pathological myopia, and there is a significant correlation between these changes and the characteristic changes associated with myopia. When diagnosing and treating pathological myopia, clinical attention should be paid to the analysis of blood flow changes behind the eyeball with the possibility of providing protection to the patient’s vision.

### Authors’ contributions:

**CY** conceived and designed the study.

**CX, ZW, XZ and XH** collected the data and performed the analysis.

**CY** was involved in the writing of the manuscript and is responsible for the integrity of the study.

All authors have read and approved the final manuscript.
